# Impact of mirna-21 on survival prognosis in patients with pancreatic cancer: A protocol for systematic review and meta-analysis

**DOI:** 10.1097/MD.0000000000022045

**Published:** 2020-08-28

**Authors:** Wei Zhang, Jing Chen, Guoqian He, Wenming Xu, Guolin He

**Affiliations:** a Department of medical oncology, Sichuan Cancer Hospital & Institute, Sichuan Cancer Center, Cancer Hospital affiliate to School of Medicine; b Key Laboratory of Birth Defects and Related Diseases of Women and Children (Sichuan University), Ministry of Education; c Department of Pediatrics, Sichuan; d Joint Laboratory of Reproductive Medicine; e Department of Obstetrics and Gynecology, West China Second University Hospital, Sichuan University, Chengdu, China.

**Keywords:** meta-analysis, miRNA-21, pancreatic cancer, prognosis

## Abstract

**Background:**

Previous studies have reported that microRNA-21 (mRNA-21) has an effect on the prognosis of pancreatic cancer. However, the conclusion is still unclear. Therefore, this study will try to explore the effect of high expression of mRNA-21 on the prognosis of pancreatic cancer.

**Methods:**

Retrieved the database, including the China National Knowledge Infrastructure (CNKI), Chinese Biomedical literature Database (CBM), Chinese Scientific and Journal Database (VIP), Wan Fang database, PubMed, and EMBASE. Hazard ratios (HRs) and its 95% confidence intervals (CIs) to assess the prognostic effect of miRNA-21 on overall survival (OS) and disease-free survival (DFS). RevMan 5.3 and STATA 16.0 software were used to perform the meta-analysis.

**Results:**

This study will comprehensively review and evaluate the available evidence of high expression of miRNA-21 on the prognosis of patients with pancreatic cancer.

**Conclusion:**

Our findings will show the effect of high expression of miRNA-21 on the prognosis of patients with pancreatic cancer. Such studies may find a new prognostic marker for patients with pancreatic cancer and help clinicians and health professionals make clinical decisions.

**Ethics and dissemination:**

The private information from individuals will not publish. This systematic review also will not involve endangering participant rights. Ethical approval is not available. The results may be published in a peer- reviewed journal or disseminated in relevant conferences.

**OSF Registration number:**

DOI 10.17605/OSF.IO/2A6KJ.

## 1 Introduction

Pancreatic cancer is one of the common malignant tumors. Its occurrence accounts for 2.2% of all cancers^[1]^ and ranks fourth among deaths caused by cancer.^[2]^ Because of the lack of early clinical manifestations, aggressive growth of tumors, and early spread of tumors, most patients are already in the advanced stage when they are diagnosed with pancreatic cancer, and therefore cannot undergo surgery.^[3]^ In addition, the tumor is also resistant to radiotherapy and chemotherapy drugs, and the 5-year survival rate is lower than that of many solid malignant tumors.^[4]^

At present, the diagnosis of pancreatic cancer mainly relies on some non-specific tumor markers, but due to their non-specificity, patients cannot be diagnosed in time and early and the disease has progressed to an advanced stage, greatly reducing the survival rate of patients. Therefore, the discovery of a new tumor marker for pancreatic cancer is very important. In recent years, with the rapid development of molecular biology, numerous studies have shown that MicroRNA (miRNA) is closely related to the occurrence, development and prognosis of tumors.^[5]^

miRNA is a type of small non-coding single-stranded RNA molecule with a length of about 22 nucleotides. It regulates the expression level of post-transcriptional genes by binding to the 3’untranslated region of target mRNA and degrades or inhibits mRNA expression.^[6]^ Many miRNAs are of great significance. Studies have shown that miRNA-21 plays a vital role in the occurrence and development of malignant tumors in the blood system and solid organs.^[7]^

Many studies have found that an important factor related to the prognosis of pancreatic cancer is the high expression of miRNA-21, but there are significant differences in research results.^[8–12]^ In order to more accurately analyze the impact of high expression of miRNA-21 on the survival of pancreatic cancer patients. This study comprehensively searched the literature related to miRNA-21 expression and the prognosis of pancreatic cancer patients, and used the method of meta-analysis to evaluate the impact of high miRNA-21 expression on the prognosis of pancreatic cancer patients.

## 2 Methods

### 2.1 Study registration

This meta-analysis protocol is based on the Preferred Reporting Items for Systematic Reviews and meta-analysis Protocols (PRISMA-P) statement guidelines.^[13]^ The PRISMA-P checklist for the protocol is provided in the PRISMAP-checklist.

The protocol of the systematic review has been registered on Open Science Framework. The registration number is DOI 10.17605/OSF.IO/2A6KJ.

### 2.2 Data sources and search strategy

We will search China National Knowledge Infrastructure (CNKI), Chinese Biomedical literature Database (CBM), Chinese Scientific and Journal Database (VIP), Wan Fang database, PubMed, and EMBASE. Search all these electronic databases without language restrictions. The detailed PubMed search strategy is in Table 1. Similar search strategies are adopted for the retrieval of other electronic databases.

**Table 1:**
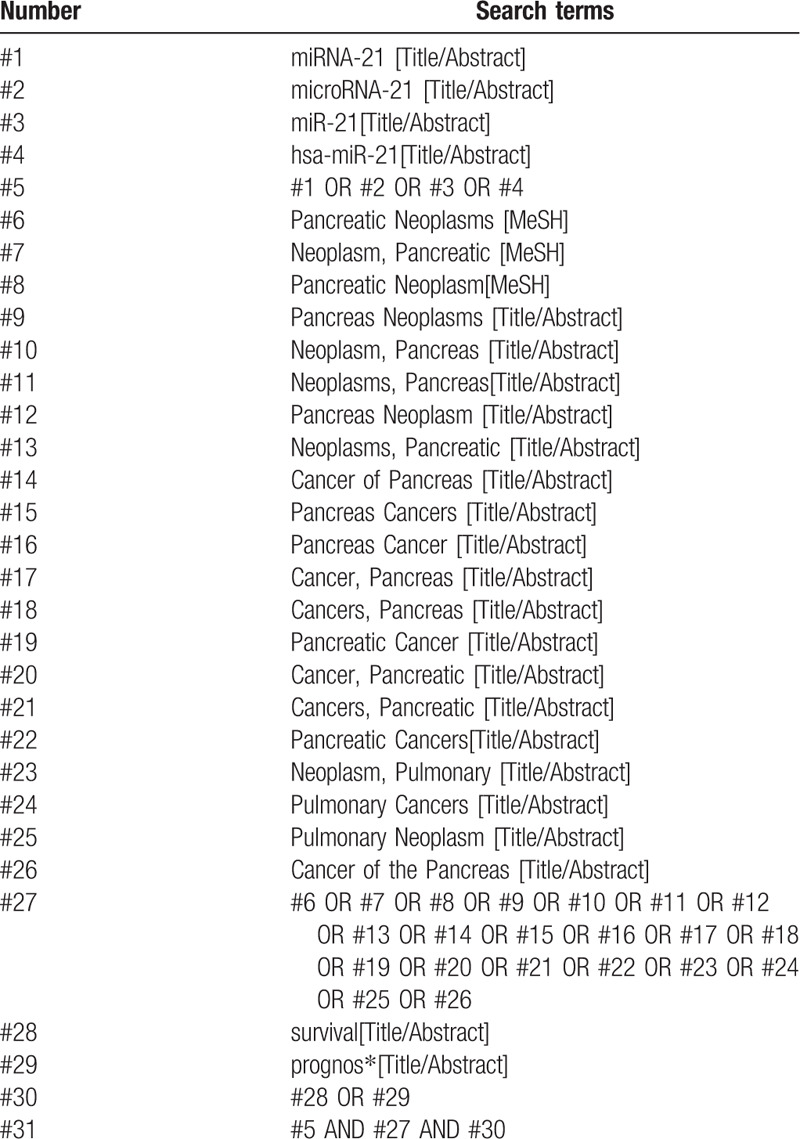
Search strategy in PubMed database. Table 1 PubMed search strategy.

### 2.3 Inclusion criteria for study selection

1. Patients diagnosed with pancreas based on pathology and histology.
2. miRNA-21 is expressed in tumor tissues.
3. Reported miRNA-21 survival-related data, including overall survival (OS) and disease-free survival (DFS).
4. Patients are divided into miRNA-21 positive (high) and miRNA-21 negative (low).
5. The article provides the relationship between miRNA-21 expression and clinical pathological characteristics.
6. Published as full-text articles, original Chinese, and English Research papers.

If there are repeated articles, choose the 1 with higher quality and larger sample size. Conference abstract, case series report, letters, animal experiments, lack of measurement indicators, and survival Research are not included.

### 2.4 Data collection and analysis

#### 2.4.1 Selection of studies

**Figure 1:**
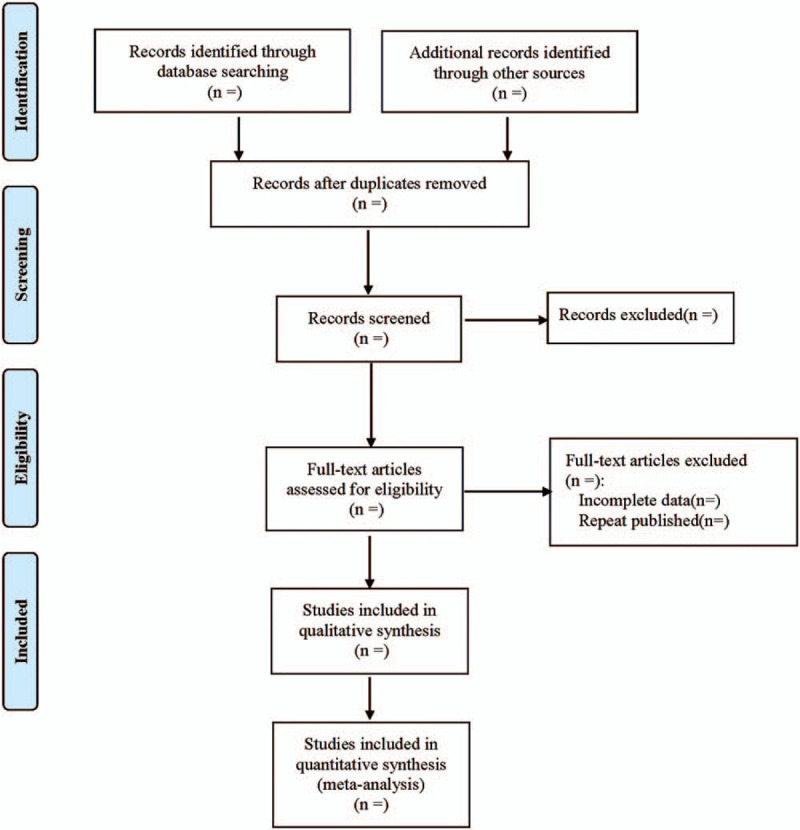
Flow diagram of study selection process.

The 2 authors will independently review the titles/abstracts of all confirmed documents, and all irrelevant studies will be excluded. Then, the full text of potentially relevant papers will be obtained to determine whether they meet all the inclusion criteria. Any differences will be resolved by consensus with the help of another experienced author. All excluded studies with detailed reasons will be recorded at different stages. The flow chart of the research selection (Fig. 1) will give a detailed description.^[13,14]^

#### 2.4.2 Data extraction and management

The 2 authors will collect information independently from each included study. Any conflicts will be resolved by consensus with the help of a third experienced author. The extracted information includes manuscript name, first author name, journal, publication year, country, race, age, gender, sample size, miRNA-21 detection method, hazard ratios (HRs), and 95% confidence intervals (CIs) of OS and DFS. We obtained HRs and 95% CIs from the Kaplan–Meier survival curves through using Engauge Digitizer version 4.1 (http://digitizer.sourceforge.net/). We will contact the lead author to obtain any missing or ambiguities from the included studies Information.

### 2.5 Assessment of quality in included studies

The quality assessment of all included studies was conducted independently by 3 researchers. The Newcastle-Ottawa Quality Assessment Scale (NOS), an observational research bias risk assessment tool recommended by Cochrane Collaboration, was used to evaluate the quality of the included studies. Any disputes are resolved through discussion. NOS consists of the following 3 quality parameters: selection, comparability, and result evaluation. Each study was scored from 0–9 according to these parameters.

### 2.6 Measures of prognosis

OS and DFS will be taken as prognostic outcomes. The results will be expressed as HRs with 95% CIs.

### 2.7 Management of missing data

If there are insufficient or missing data in the literature, we will contact the author via email to request data. If the data is not available, we will only analyze the currently available data and discuss its potential value.

### 2.8 Statistical analysis

The 95% CIs and HRs were used to evaluate the relationship between miRNA-21 expression and OS and DFS. Statistical heterogeneity tests were performed on the included studies. If there is no statistical heterogeneity among the included literatures (*I*^*2*^ ≤ 50%, *P* < .1), a fixed effect model is used; when there is statistical heterogeneity among the included literatures (*P* < .1, *I*^*2*^ ≥ 50%), the sources of heterogeneity will be analyzed. Clinical heterogeneity will be treated by subgroup analysis. In the absence of significant clinical heterogeneity and methodological heterogeneity, statistical heterogeneity will be considered, and a random effects model will be used for analysis. If the clinical heterogeneity of the subgroup analysis is significantly higher, no meta-analysis will be performed and only a descriptive analysis will be performed. Statistical analysis was performed using STATA 14.0 (STATA Corporation, College Station, TX, USA) and RevMan 5.3 (The Nordic Cochrane Centre, The Cochrane Collaboration, 2014).

### 2.9 Additional analysis

#### 2.9.1 Subgroup analysis

We conduct subgroup analysis based on race, miRNA-21 detection method, survival data source, and miRNA-21 high expression threshold.

#### 2.9.2 Sensitivity analysis

We will use the one-by-one elimination method to analyze the sensitivity of each indicator to test the stability of the meta-analysis results.

#### 2.9.3 Reporting bias

If the number of studies included in a certain outcome index is no less than 10, funnel chart is used to evaluate publication bias. Besides, Eggers and Beggs test were used for the evaluation of potential publication bias.

## 3 Discussion

Pancreatic cancer lacks specific clinical symptoms and is often in the advanced stage of the disease when it is diagnosed. It is one of the deadliest cancers and extremely aggressive and lacks reliable early diagnostic markers.^[14,15]^ The current status of pancreatic cancer treatment is not optimistic. The 5-year survival rate of patients is less than 5%.^[16]^ Therefore, in-depth exploration of the pathogenesis of pancreatic cancer can provide experimental basis for formulating treatment strategies and has important research significance. There is important research significance to explore the diagnostic and predictive markers of pancreatic cancer, which can provide experimental basis for formulating treatment strategies.

There are many methods for clinical diagnosis of pancreatic cancer, including serological tumor marker examination, radiological examination, and pathological examination et al.^[17]^ These inspection methods are of great help to the diagnosis and tumor location of pancreatic cancer. Nevertheless, it is not difficult to find that these inspection methods themselves have certain defects, such as poor specificity and low sensitivity.^[18,19]^

miRNA is a type of non-coding RNA with a length of about 22 nt, which is involved in the regulation of the development, cell growth, and differentiation of many eukaryotic organisms.^[20]^ Abnormal expression of miRNA is associated with the progression of a variety of tumors.^[21]^ miRNA-21 has a variety of biological effects^[21]^ and its role in malignant tumors is one of the research hotspots. The expression level of miRNA21 can increase by 5 to 100 times with the increase of the malignant degree of human glioblastoma. miRNA-21 can be used as a diagnostic marker for human glioblastoma.^[22,23]^ In addition, miRNA21 is widely involved in the occurrence and development of breast cancer, colon cancer, and pancreatic cancer.^[24]^ Among them, miRNA-21 shows a high expression trend in the early stage of pancreatic cancer differentiation,^[25]^ and which can regulate cell proliferation, invasion, and apoptosis.^[26]^ And the expression level of miRNA-21 is related to the degree of differentiation of tumor cells, and negatively related to the prognosis of patients.^[27]^ However, there is currently no convincing evidence that high expression of miRNA-21 has an effect on the prognosis of pancreatic cancer. Therefore, we tried to analyze and summarize the impact of miRNA-21 on the prognosis of pancreatic cancer through this meta-analysis.

Several limitations existed in our study. Firstly, the cut-off values for miRNA-21 expression measured by the different method varied between studies, which may cause heterogeneity in the overall results. Secondly, the inclusion of ethnic groups in our study may be incomplete, and whether the conclusion can guide other ethnic groups is unknown. In addition, the treatments taken by patients before and after surgery are unclear, so it has a certain impact on the quantitative analysis of prognosis in this meta-analysis. Lastly, not all HRs and 95% CIs were directly extracted from the studies, as we had to extract some data using Kaplan–Meier curves, which nay influence the precision of the data.

## 4 Conclusion

This meta-analysis will provide more accurate and objective evidence for the relationship between high miRNA-21 expression and pancreatic cancer survival.

## Author contributions

**Conceptualization**: Wei Zhang and Guolin He.

**Data collection**: Jing Chen and Guoqian He.

**Funding acquisition**: Jing Chen and Guolin He.

**Resources**: Wenming Xu.

**Software**: Wenming Xu.

**Supervision**: Wenming Xu and Jing Chen.

**Writing** – **original draft**: Wei Zhang and Guolin He.

**Writing** – **review & editing**: Wei Zhang and Guolin He.

## Correction

Two references, 13 and 14, have been added to the last sentence of 2.4.1. The reference list has been updated to reflect this addition.
